# Distinguishing cerebrospinal fluid from mepivacaine using the pH test in patients undergoing elective cesarean section with combined spinal-epidural anesthesia

**DOI:** 10.1186/s40981-020-00383-y

**Published:** 2020-10-02

**Authors:** Hiromi Ikegami, Kunihisa Hotta, Yoshie Toba

**Affiliations:** 1 Department of Anesthesiology, Seirei Hamamatsu General Hospital, 2-12-12 Sumiyoshi, Naka-ku, Hamamatsu city, Shizuoka, 430-8558 Japan; 2 Department of Anesthesiology and Critical Care Medicine, Jichi Medical University, Tochigi, Japan

**Keywords:** Cerebrospinal fluid, Acid-base equilibrium, Epidural anesthesia, Spinal anesthesia, Mepivacaine

## Abstract

**Introduction:**

In single-space combined spinal-epidural anesthesia (CSEA), it is important to correctly determine if the fluid coming out of the spinal needle is cerebrospinal fluid (CSF) or the liquid used in the loss of resistance (LOR) technique. In this study, we used mepivacaine for LOR and measured the pH values of CSF and mepivacaine to determine whether the pH test is a reliable method to confirm CSF when performing single-space CSEA.

**Methods:**

This clinical trial included 47 full-term pregnant women who underwent cesarean section. Single-space CSEA was administered at the lumbar intervertebral space using a small amount of mepivacaine for LOR. The pH values of CSF and mepivacaine were determined by the color of the test strip immediately after dropping. The area under the curve (AUC) for the pH values was calculated to determine the cutoff value for distinguishing between CSF and mepivacaine.

**Results:**

The median pH values were 7.7 (7.1–8.0) and 6.2 (5.9–6.8) for CSF and mepivacaine, respectively. When the cutoff value of pH for distinguishing CSF from mepivacaine was 7.1 or greater, the AUC was 1.0 (100% sensitivity and specificity). Our result demonstrated that CSF can be correctly distinguished from mepivacaine in patients undergoing cesarean section under single-space CSEA using a cutoff value of pH 7.1.

**Conclusion:**

The pH test is a simple and reliable method to confirm CSF when performing single-space CSEA with mepivacaine for LOR.

**Trial registration:**

Accuracy of pH test paper for cerebrospinal fluid during spinal anesthesia: prospective study in healthy pregnant women under scheduled caesarean section; University Hospital Medical Information Network, UMIN000036454. Registered 1 May 2019

## Background

Combined spinal-epidural anesthesia (CSEA) is widely used for cesarean sections due to its advantage of long-lasting postoperative analgesia. When single-space CSEA is performed, the epidural space is identified by the loss of resistance (LOR) technique using saline. Subsequently, the subarachnoid space is punctured using a spinal needle inserted through the epidural needle, and the local anesthetic is administered intrathecally. Since the saline used in the LOR technique may come out of the spinal needle, care should be taken to ensure that the liquid is cerebrospinal fluid (CSF) for successful anesthesia.

The qualitative sugar test is a convenient technique for confirming CSF that provides quick results in 30–60 s. However, small traces of blood in the CSF can deem the sugar test positive. Once a vascular puncture occurs during needle insertion, it is difficult to confirm CSF in a sugar test. Therefore, this test may not be suitable for confirming CSF in pregnant women with a dilated epidural venous plexus.

The pH test is another simple technique for confirming CSF. Previous studies showed the pH test to be as useful as the sugar test [1, 2]. Since the pH value of CSF does not change with a small amount of blood, the pH test is less sensitive to blood contamination than the sugar test. However, the pH test has not been commonly used for CSF confirmation, probably because the pH values of CSF and saline (CSF pH, 7.28–7.39; saline pH, 4.6–8.0) are overlapped [3–5]. We came up with the idea that a small amount of mepivacaine (pH, 4.5–6.8) could be used for the LOR technique when confirming CSF using pH paper [6].

In this study, we used mepivacaine for LOR and measured the pH values of CSF and mepivacaine to determine whether the pH test is a reliable method for confirming CSF when performing single-space CSEA. We also determined a cutoff value of pH to distinguish between CSF and mepivacaine.

## Methods

The study was approved by the Institutional Ethics Committee (#3040) on April 16, 2019, and was registered at the University Hospital Medical Information Network (ID UMIN000036454) on May 1, 2019. Written informed consent was obtained from all participants. This study was conducted at the Seirei Hamamatsu General Hospital in Shizuoka, Japan, between May 2019 and July 2019.

We included pregnant women aged > 20 years who were scheduled for elective cesarean section with single-space CSEA. Parturients with an American Society of Anesthesiologists physical status ≥ 3 and those whose native language was not Japanese were excluded. Demographic data, including the patient’s age, gestational week, and the indication for cesarean section were obtained from the medical records.

**Fig. 1:**
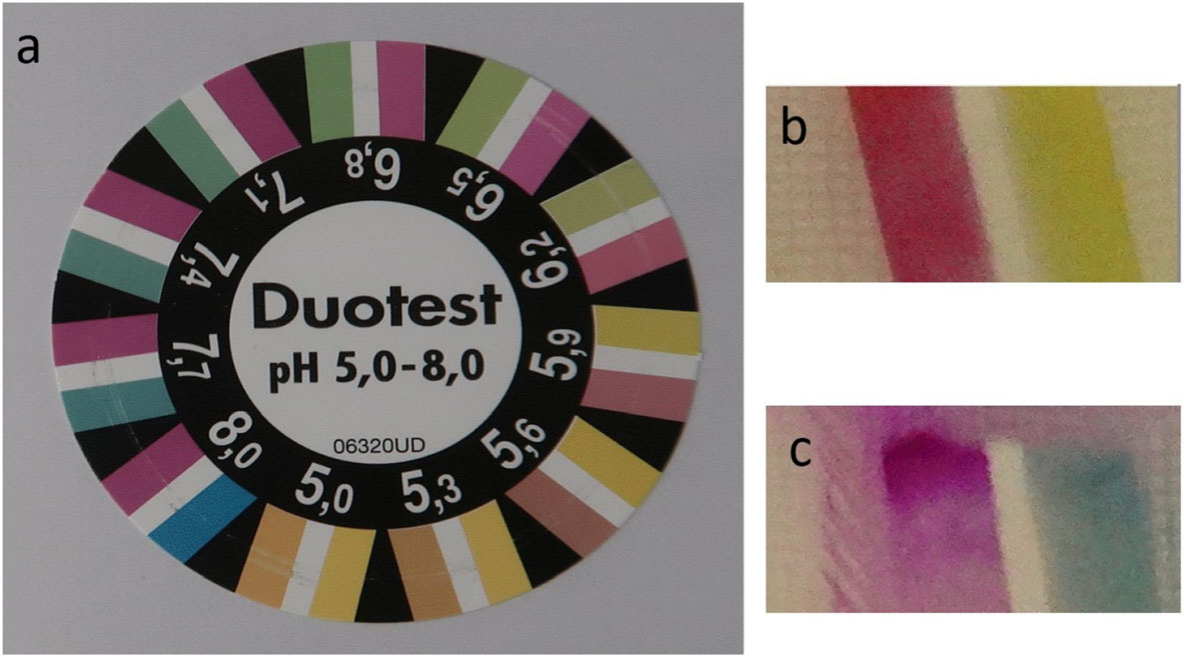
pH test paper. **a** Duotest pH 5.0–8.0®, MACHEREY-NAGEL GmbH & Co. KG. These indicator papers show two different colors for each pH value at intervals of 0.3–1 pH units. This allows for more accurate reading and a good estimation of intermediate values. The two sidebands are indicators and change color. The white center band separates the above two bands to avoid fusion of the indicator dyes. The pH value of the target sample is decided by comparing its color with this color chart. **b** Mepivacaine on a pH test paper. It is a representative sample of mepivacaine determined to have a pH of 6.2. **c** Cerebrospinal fluid on a pH test paper. The upper part of the figure corresponds to the center of **a**. It is a representative sample of CSF determined to have a pH of 7.7

In the operating room, single-space CSEA was administered at the L2/3, L3/4, or L4/5 interspace with the parturient in the lateral decubitus position. Oxygen saturation, non-invasive blood pressure, and electrocardiogram were continuously monitored for all patients. After local infiltration with 1% mepivacaine (mepivacaine hydrochloride injection syringe, Maruishi Pharmaceutical Co., Ltd, Osaka, Japan), single-space CSEA was performed using a combined spinal-epidural needle (Portex® CSEcure® spinal-epidural system 16G–27G, Smiths Medical, Tokyo, Japan). The epidural space was identified using the LOR technique with a small amount of 1% mepivacaine. After confirming that there was no dural puncture, the spinal needle was passed through the epidural needle and advanced to the subarachnoid space. When a clear liquid came out of the spinal needle, it was dropped onto a pH paper (Duotest pH 5.0–8.0®, MACHEREY-NAGEL GmbH & Co. KG, Düren, Germany) placed on a white gauze. The pH values were determined under fluorescent light by two members of the medical staff in the operating room, where they indicated the most complementary color to that of the pH test paper (Fig. 1a). The pH values of the residual mepivacaine in the LOR syringes were also determined using the pH paper. The measured sample was determined as CSF when a reasonable anesthetic level was obtained.

The primary outcome measure was the cutoff value of pH that distinguishes CSF from mepivacaine. The secondary outcome measure was the reliability of the judged pH value between the two evaluators. Perioperative complications, such as respiratory depression (requiring assisted ventilation), post-dural puncture headache (PDPH), and nerve injury, were also assessed.

The sample size was calculated based on a two-sided test with the area under the curve (AUC) of 0.75, a power of 0.9, and a significance level of 5%. The determined required sample size was 25. Statistical analysis was performed using computer software R (version 3.6.1, The R Foundation for Statistical Computing, Vienna, Austria). The AUC for the pH values was calculated, and the cutoff value was decided by the intersection of the diagonal line and the receiver operating curve. The AUC takes a value of 0.5–1.0, with values 0.9–1.0 indicating a high predictive ability. The reliability of the pH test for identification of both CSF and mepivacaine was assessed using Cohen’s weighted kappa coefficient. The *κ* coefficient takes a value of 0–1, with values greater than 0.6 indicating substantial agreement.

## Results

Of the 51 eligible participants, three were excluded because their native language was not Japanese, and one patient did not provide consent. Thus, 47 pregnant women with a mean age of 34.7 ± 5.0 years and a mean gestational age of 37.9 ± 0.5 weeks were included in this study. The indications for cesarean section were past cesarean section (*n* = 26), breech presentation (*n* = 8), twins (*n* = 4), placental abnormalities (*n* = 4), and others (*n* = 5).

The median pH values were 7.7 (range, 7.1–8.0) and 6.2 (range, 5.9–6.8) for CSF and mepivacaine, respectively (Fig. 1b, c). When the cutoff value of pH for distinguishing CSF from mepivacaine was 7.1 or greater, the AUC was 1.0 (100% sensitivity and specificity). The values of the weighted kappa coefficient for assessing the agreement between the two evaluators on the pH values for identification of CSF and mepivacaine were 0.65 and 0.76, respectively. CSEA was successful in all participants, and all samples were determined as CSF. There were no registered complications of respiratory depression (requiring assisted ventilation), PDPH, or nerve injury.

## Discussion

The results of the present study demonstrate that CSF can be correctly distinguished from mepivacaine based on the pH test with a cutoff value of pH 7.1. The weighted *κ* coefficients were greater than 0.6, the pH tests judged substantially matches.

Previous studies have demonstrated the superiority of the sugar test over the pH test [1, 2]. Since artificial CSF was used in these studies, there were no false positives due to blood contamination in the sugar test. However, the pH paper used in this study could identify a small pH difference of 0.3 compared to that used in the previous studies that had a scale of 1. Moreover, the use of mepivacaine for the LOR technique in our study may have contributed to a more accurate pH assessment compared with that based on the use of saline due to its high sensitivity and specificity. Accordingly, the pH test appears to be a reliable and convenient technique for identifying CSF.

When performing our single-space CSEA technique, mepivacaine was used for the LOR instead of saline or air. Mepivacaine was considered to be advantageous over saline for distinguishing it from CSF by pH value. The use of air for the LOR technique has a serious complication of pneumocephalus. On the other hand, the use of a local anesthetic for the LOR technique may provoke high spinal block and unintentional intrathecal administration of the local anesthetic. Previous studies have shown that the extent and duration of the spinal block were similar with or without epidural volume extension by 5 ml of saline or 10 ml of 0.25% bupivacaine [7, 8]. Intrathecal administration of 3 ml of mepivacaine 2% (60 mg) to parturients undergoing cesarean section resulted in adequate sensory blockade with a median sensory block height of T5 [9]. Besides, although intrathecal administration of mepivacaine has a risk of transient neurologic symptoms (TNS), the incidence of TNS after spinal anesthesia with mepivacaine was much lower than that with lidocaine [10]. However, it is safer to perform the LOR technique with a smaller and lower concentration of local anesthetic. To minimize the risk of unintentional intrathecal administration of local anesthetic during the LOR technique, all participating anesthesiologists had over 10 years of experience and used a small amount of local anesthetic, less than 3 ml of 1% mepivacaine. None of the parturients showed signs of high spinal block and TNS. In addition, mepivacaine was used for both local infiltration and LOR in the present study. A case of mepivacaine-induced anaphylactic shock has recently been reported [11]. Therefore, the use of mepivacaine has a potential risk of an allergic reaction.

The determination of CSF with pH test may be useful not only for single-space CSEA, but also when local anesthetics are administered into the epidural space prior to spinal anesthesia. When spinal anesthesia is required for cesarean section during labor epidural analgesia, a large amount of local anesthetic administered into the epidural space may cause incorrect assessment for subarachnoid puncture. Ropivacaine is commonly used for labor epidural analgesia, and its pH value (4.0–6.0) does not overlap with that of CSF [12]. Future studies to assess the feasibility of CSF confirmation with the pH test in this clinical setting may be informative.

The limitation of this study was that the fluid obtained from the spinal needle was CSF in all cases. We did not measure the pH value of mepivacaine administered into the epidural space by the LOR. The cutoff value of pH to distinguish between CSF and mepivacaine in non-pregnant patients may differ from our findings.

## Conclusions

The pH test is a simple and reliable method to distinguish between CSF and mepivacaine when performing single-space CSEA, and the cutoff value of pH was determined to be 7.1.

## Data Availability

The datasets used and/or analyzed during the current study are available from the corresponding author (HI) on reasonable request.

## Abbreviations

AUC: Area under the curve
CSEA: Combined spinal-epidural anesthesia
CSF: Cerebrospinal fluid
LOR: Loss of resistance
PDPH: Post-dural puncture headache
TNS: Transient neurologic symptoms
